# PERSON-CENTERED DEMENTIA TRAINING ONLINE FOR BSN STUDENTS: A PILOT STUDY

**DOI:** 10.1093/geroni/igad104.3521

**Published:** 2023-12-21

**Authors:** Diana Sturdevant, Kimetha Broussard, Keith Kleszynski, Lee Jennings

**Affiliations:** University of Oklahoma HSC, Fran and Earl Ziegler College of Nursing, Oklahoma City, Oklahoma, United States; University of Oklahoma HSC, Fran and Earl Ziegler College of Nursing, Oklahoma City, Oklahoma, United States; University of Oklahoma Health Sciences Center, Oklahoma City, Oklahoma, United States; University of Oklahoma Health Sciences Center, Oklahoma City, Oklahoma, United States

## Abstract

We launched a Person-Centered Dementia Training in March 2023, focused on Four interactive modules were offered via an Online Learning Platform. Topics included: Introduction to Dementia, Communication Process and Approach, Environmental and Cultural Implications in Dementia Care, and Best Practices in Responding to Difficult Behaviors. Subtopics included: Introduction to Age-Friendly and Dementia-Friendly Care, High-risk Medications and Dementia Care, Dementia vs Delirium, and Reframing Aging—Understanding and Recognizing Ageism Specific to People Living with Dementia. BSN students were recruited via (3) email blasts and (1) in-person announcement of participation opportunity. A total of (n=33) participants were recruited from 4 nursing programs, Traditional BSN Junior (34%), Traditional BSN Senior (48%), Degree Completion LPN to BSN (3%) and Accelerated BSN (14%). Most participants were female (96%), between the ages of 18-24 (60%). 24 participants completed the pre-/post-test and Final Program Evaluation. Participants rated the course content and structure as 4.5/5 on 5pt-likert scale. Common themes from evaluations included: need for additional content, more interactive activities, and inclusion as a required course for all nursing students. Future steps to include revising course content based on participant feedback, offer revised course over the next 2 academic semesters, and expand reach by offering course to practicing nurses to improve care of people living with dementia (PLWD).

